# Clinical indicators, nursing diagnoses, and mortality risk in critically ill patients with COVID-19: a retrospective cohort

**DOI:** 10.1590/1980-220X-REEUSP-2021-0568en

**Published:** 2022-07-01

**Authors:** Elis Maria Secoti Barioni, Cawana da Silva do Nascimento, Thatiana Lameira Maciel Amaral, José Melquíades Ramalho, Patrícia Rezende do Prado

**Affiliations:** 1 Universidade Federal do Acre, Curso de Bacharelado em Enfermagem, Rio Branco, AC, Brazil.; 2 Universidade Federal do Acre, Residência Multiprofissional em Terapia Intensiva, Rio Branco, AC, Brazil.; 3 Universidade Federal do Acre, Programa de Pós-Graduação em Saúde Coletiva, Residência Multiprofissional em Terapia Intensiva, Rio Branco, AC, Brazil.; 4 Universidade Federal da Paraíba, Hospital Universitário Lauro Wanderley, João Pessoa, PB, Brazil.

**Keywords:** Coronavirus, Signs and Symptoms, Nursing Diagnosis, Risk Factors, Mortality, Patient Care Planning, Coronavirus, Signos y Síntomas, Diagnóstico de Enfermería, Factores de Riesgo, Mortalidad, Planificación de Atención al Paciente, Coronavirus, Sinais e Sintomas, Diagnóstico de Enfermagem, Fatores de Risco, Mortalidade, Planejamento de Assistência ao Paciente

## Abstract

**Objective:**

To identify clinical indicators and nursing diagnoses with the highest risk of mortality in critically ill patients with COVID-19.

**Method:**

Retrospective cohort with the population of adults and elderly people with COVID-19 from an Intensive Care Unit. Categorical variables were described using absolute and relative frequencies and risk factors for mortality using Cox regression, with a confidence interval of 95%.

**Results:**

The main clinical indicators of COVID-19 patients were dyspnea, fever, fatigue, cough, among others, and the Nursing Diagnoses at higher risk of mortality were Ineffective protection, Ineffective tissue perfusion, Contamination, Ineffective Breathing Pattern, Impaired spontaneous ventilation, Acute confusion, Frailty syndrome, Obesity, and Decreased cardiac output. It is worth mentioning that there was little information about the diagnoses of Domains 9, 10, and 12.

**Conclusion:**

This research infers the need to monitor the clinical indicators dyspnea, fever, fatigue, cough, among others, and the Nursing Diagnoses with the highest risk of mortality Ineffective protection, Ineffective tissue perfusion, Contamination, Ineffective Breathing Pattern, Impaired spontaneous ventilation in critically ill patients.

## RESUMO

**Objetivo:**

Identificar os indicadores clínicos e os diagnósticos de enfermagem com maior risco de mortalidade em pacientes críticos com COVID-19.

**Método:**

Coorte retrospectiva com a população de adultos e idosos com COVID-19 de uma Unidade de Terapia Intensiva. As variáveis categóricas foram descritas por frequências absoluta e relativa e os fatores de risco para mortalidade, pela regressão de Cox, com intervalo de confiança de 95%.

**Resultados:**

Os principais indicadores clínicos de pacientes com COVID-19 foram dispneia, febre, fadiga, tosse, entre outros, e os Diagnósticos de Enfermagem de maior risco de mortalidade: Proteção ineficaz; Perfusão tissular ineficaz; Contaminação; Padrão Respiratório Ineficaz; Ventilação espontânea prejudicada; Confusão aguda; Síndrome do idoso frágil; Obesidade e Débito cardíaco diminuído. Vale ressaltar que havia poucas informações sobre os diagnósticos dos Domínios 9, 10 e 12.

**Conclusão:**

Esta pesquisa infere a necessidade de vigiar os indicadores clínicos dispneia, febre, fadiga, tosse, entre outros e os Diagnósticos de Enfermagem de maior risco de mortalidade Proteção ineficaz; Perfusão tissular ineficaz; Contaminação; Padrão Respiratório Ineficaz; Ventilação espontânea prejudicada em pacientes críticos.

## RESUMEN

**Objetivo:**

Identificar los indicadores clínicos y los diagnósticos de enfermería con más riesgo de mortalidad en pacientes críticos con COVID-19.

**Método:**

Cohorte retrospectiva con la población de adultos y ancianos con COVID-19 de una Unidad de Cuidados Intensivos. Las variables categóricas fueron descriptas por frecuencias absoluta y relativa y los factores de riesgo para mortalidad, por la regresión de Cox, con intervalo de confianza del 95%.

**Resultados:**

Los principales indicadores clínicos de pacientes con COVID-19 fueron disnea, fiebre, fatiga, tos, entre otros y los Diagnósticos de Enfermería de mayor riesgo de mortalidad: Protección ineficaz; Perfusión tisular ineficaz; Contaminación; Patrón Respiratorio Ineficaz; Ventilación espontánea perjudicada; Confusión aguda; Síndrome del anciano frágil; Obesidad y Débito cardíaco disminuido. Se puede destacar que había pocas informaciones sobre los diagnósticos de los Dominios 9, 10 y 12.

**Conclusión:**

Esa investigación implica que hay necesidad de monitorear los indicadores clínicos disnea, fiebre, fatiga, tos, entre otros y los Diagnósticos de Enfermería con más riesgo de mortalidad, Protección ineficaz; Perfusión tisular ineficaz; Contaminación; Patrón Respiratorio Ineficaz; Ventilación espontánea perjudicada en pacientes críticos.

## INTRODUCTION

In the International Year of the Nurse, 2020, nurses around the world faced a great professional challenge to care for patients with the disease caused by the new coronavirus, COVID-19, which emerged from an epidemic in China, and is characterized by high transmissibility and morbidity and mortality^(1)^.

On December 3, 2021, it had already affected more than 262 million people, with more than five million dying. The most affected continent is the American, with more than 97 million cases. In Latin America, Brazil was the second country with the highest confirmed incidence and mortality, with more than 22 million cases and 614,000 deaths, whose risk factors for mortality shall be investigated aiming at the early identification of clinical indicators present in patients with COVID-19^(2,3)^.

Thus, in the context of the COVID-19 pandemic, health care services have demanded rapid and systematic assistance from the Nursing team, whose actions are related to the Nursing Process (NP), which is the scientific method used by the nurse to identify, plan, intervene, and assess care^(3)^.

The NP highlights the Nursing contribution to the population’s health care, increasing visibility and professional recognition. The NP is subsidized by Resolution No. 358 of 2009, of the Federal Nursing Council (COFEN) and consists of five stages: Data/History Collection, Nursing Diagnosis, Planning, Implementation, and Assessment^(4,5)^.

The Nursing History is NP’s first stage and consists of the patient’s history taking and physical examination. At this stage, the nurse identifies symptoms and signs, which can also be called clinical indicators/clinical manifestations, which will serve to prepare the Nursing Diagnoses (ND). The NDs are Nursing problems identified through History taking that they need to receive Nursing interventions, and in case of the presence of associated conditions, interventions from other members of the interdisciplinary team^(4)^. The NDs are listed in Nursing taxonomies, with the NANDA-*international*, Inc. (NANDA-I) being chosen for this research, due to the greater familiarity of the researchers^(4,5)^.

In patients with COVID-19, it has been reported that the main symptoms and signs of the disease are fever, cough, dyspnea, pulmonary auscultation with adventitious sounds, myalgia, running nose, diarrhea and anosmia, whose clinical indicators can infer NDs, such as impaired spontaneous ventilation, impaired gas exchange, ineffective peripheral tissue perfusion, risk of pressure injury, risk of corneal injury, among others, as described by the Nursing Process Research Network (*RePPE*), in its tutorial and data collection instrument for the elaboration of Nursing diagnoses, outcomes, and interventions for the care of patients in critical condition during the COVID-19 pandemic^(6–10)^. However, these suggested nursing diagnoses have not been clinically validated, and there may be others.

Moreover, several articles reported that age over 60 years, male sex, presence of chronic diseases, and unhealthy lifestyle are higher risk factors for morbidity and mortality in patients with COVID-19 and, in the context of NDs, these factors are called risk/related factors and associated conditions, whose interventions need the help of other professionals for their resolution^(5–10)^.

In COVID-19, respiratory manifestations have been emphasized for clinical observation, such as increased respiratory rate, dyspnea, use of accessory muscles, low oxygen saturation, among others, which can suggest the NDs Ineffective Breathing Pattern (IBP), Impaired Gas Exchange and Impaired Spontaneous Ventilation, for example, whose care interventions will be directed to dyspnea relieve, correcting acid-base imbalances (especially respiratory acidosis) and establishing normal respiratory function^(10–12)^.

Thus, it is thought that identifying the Nursing diagnoses with a higher risk of mortality in patients with COVID-19 can help in the surveillance of clinical indicators and in the early targeting of interventions aimed at patients’ recovery. In this regard, the question of this study is: What are the clinical indicators and nursing diagnoses with the highest risk of mortality in critically ill patients with COVID-19?

To answer the study question, the objective of this work is to identify clinical indicators and nursing diagnoses with the highest risk of mortality in critically ill patients with COVID-19.

## METHOD

### Design of Study

This is a retrospective cohort study with patients hospitalized due to COVID-19 complications, in an Intensive Care Unit, from March to December 2020.

### Population, Local and Selection Criteria

The sample consisted of all (57) adult and elderly patients, diagnosed with COVID-19, who were hospitalized in the COVID-19 ICU of a hospital in the Brazilian Amazon, for at least 72 hours, and who died. The information was collected from the patients’ clinical records, at the Medical Archives Service (*SAME*), from the admission to the discharge/death of the patient in the ICU. Data collection was carried out from January to September 2021.

### Dependent Variable

The dependent variable was death of patients with COVID-19.

### Independent Variables

The independent variables were the sociodemographic, the clinical ones, and the NDs, with the first two being variables that are ND’s clinical indicators (defining characteristics).

The sociodemographic variables listed were: medical record number, date of birth, date of onset of symptoms, date of diagnosis, date of discharge or death, length of stay (days), age (years) and color (white, brown, yellow or black), with the last two factors being related to possible ND.

The NDs were selected from the NANDA-I taxonomy, collected in a dichotomous way and discriminated by presence or absence, according to what was established in the ICU-COVID-19 NP instrument.

The defining characteristics (which are the clinical manifestations, symptoms and signs – clinical indicators) considered were: fever; cough; dyspnea; tachypnea; tachycardia; pulmonary auscultation with adventitious sounds; acid-base imbalance; myalgia; running nose; diarrhea; anosmia; pain; pure ground-glass opacity with or without consolidation; weakened breath sounds; dullness on percussion and increase or decrease in tactile speech tremor; nasal flaring; use of accessory muscles; breathing with pursed lips; increased anteroposterior diameter of the chest; hypoxia; restlessness; headache when waking up; diaphoresis; abnormal skin color; somnolence; hypovolemia; hematuria; proteinuria; dehydration; and abnormal levels of serum electrolytes^(5,7,8,12,13)^.

The risk actors and associated conditions collected were: age; heart diseases; lung diseases; depressed immune system; obesity; diabetes; chest wall deformity; kidney and liver diseases; weight; height; and body mass index^(5,7,8,12,13)^.

### Data Collection Procedure

Data were collected using a questionnaire created digitally in the software *Research Electronic Data Capture* (REDCap)^(14)^.

REDcap was created in 2004 by researchers at *Vanderbilt University* (Tennessee, United States). It has the financial support of the *National Institute of Health (NIH),* and has technical-scientific support from the *REDCap Consortium*, made up of more than 2,600 institutions in more than 117 countries on six continents. It was introduced in Brazil in 2011 through the Medical School of Universidade de São Paulo (FMUSP). Currently, the REDCap Brasil Consortium is the entity in the country responsible for the official representation of the tool at more than 100 renowned institutions^(14)^.

The data collection procedure for the research was performed by a nursing resident of the Multiprofessional Residency Program in Intensive Care at the Universidade Federal do Acre.

### Data Analysis and Treatment

The analysis was performed with the software SPSS, version 22.0. Continuous variables were analyzed by measures of central tendency (minimum, maximum, mean, and standard deviation) and categorical variables by absolute and relative frequencies. The NDs with mortality risk were identified by Cox regression, using the magnitude measure *Hazard Ratio* (HR), considering a confidence interval of 95%.

### Ethical Aspects

This project was approved by the Research Ethics Committee of Fundação Hospital Estadual do Acre (FUNDHACRE), through Opinion No. 4.429.703, on November 30, 2020. Patients or guardians authorized the research by signing the Consent Form/Assent, and received a copy.

## RESULTS

Of the 57 patients analyzed, 50.8% were female, 50.0% were married, 50.0% were over 60 years of age, 42.5% were brown, 52.6% were hypertensive, 40.3% were diabetic, and 17.5% had cardiorespiratory diseases, which were the main sociodemographic characteristics found (Table 1).

**Table 1.** Sociodemographic and clinical characteristics of critically ill patients with COVID-19. Rio Branco, AC, Brazil, 2020–2021.

Variable	N	%
Total	57	100.0
**Sex**		
Female	29	50.8
Male	28	49.2
Age*		
More than 60 years	27	50.0
Less than 60 years	27	50.0
Education*		
Illiterate up to high school	7	70.0
Finished undergraduation	3	30.0
Color*		
White	6	12.8
Brown	20	42.5
Black	1	2.2
Yellow	20	42.5
**Origin**		
Clinical emergency room	7	12.3
Transfer	50	87.7
**Readmission**		
No	57	100.0
Comorbidity**		
Systemic arterial hypertension	30	52.6
Diabetes mellitus	23	40.3
Cardiorespiratory diseases	10	17.5
Neurological disorder	02	3.5
Obesity	02	3.5
Cancer	01	1.7

*Missings. ** May have more than one comorbidity.

Regarding the clinical condition at ICU admission, 53.6% of the patients had dyspnea (RR ≥ 21 mpm) and 35.0% had O_2_ saturation ≤93%. Of the 15 (26.3%) patients who underwent chest tomography on admission, ten (66.7%) had a ground-glass pattern and five (33.3%) had more than 30.0% pulmonary involvement. The electrocardiogram (ECG) showed alterations in 92.6% of the patients, 79.0% were under sedoanalgesia, 75.4% used vasoactive drugs, and 79.0% were enterally fed (Table 2).

**Table 2.** Clinical characteristics of admission of patients with COVID-19 – Rio Branco, AC, Brazil, 2020–2021.

Variable	N	%
**Mean blood pressure**		
≤69 mmHg	06	10.5
70 to 89 mmHg	24	44.1
≥90 mmHg	27	47.4
Respiratory rate*		
12 to 20 mpm	26	46.4
≥21 mpm	30	53.6
**Heart rate**		
52 to 100 bpm	31	54.4
101 to 160 bpm	26	45.6
**Axillary temperature**		
<36°C	20	35.0
36° to 37.6°C	31	54.4
≥37.8°C	6	10.6
**Oxygen saturation (O_2_)**		
≤93%	20	35.0
>94%	37	65.0
End expiratory pressure (PEEP)*		
<8	05	11.4
≥8	39	88.6
**Use of sedoanalgesia**		
No	12	21,0
Yes	45	79.0
Types of sedoanalgesia*		
Dormonid	4	9.0
Fentanyl	1	2.0
Dormonid + Fentanyl	36	80.0
Precedex	4	9.0
**Use of vasoactive drugs**		
No	14	24.6
Yes	43	75.4
**Diet**		
Zero diet	4	7.0
Oral route	8	14.0
Oral + enteral route	45	79.0
Chest tomography*		
Ground glass pattern	10	66.7
>30% lung involvement	5	33.3
Electrocardiogram*		
Normal	2	7.4
Changed	25	92.6
Clinical indicators related to COVID-19**		
Dyspnea	46	80.7
Fever	26	45.6
Fatigue	14	24.6
Cough	13	22.8
Headache	9	15.8
Myalgia	5	8.7
Loss of appetite	1	1.75
Anosmia	1	1.75

*Missings. ** It can present more than one manifestation.

The main clinical indicators of COVID-19 were: dyspnea (80.7%), fever (45.6%), fatigue (24.6%), and cough (22.8%), in addition to headache, myalgia, loss of appetite, and anosmia.

The NDs with the highest risk of mortality were: Ineffective protection; Ineffective tissue perfusion; Contamination; Ineffective Breathing Pattern; Impaired spontaneous ventilation; Acute confusion; Frailty syndrome; Obesity, and Decreased cardiac output, all of them with 95% CI. It is worth mentioning that there was little information about the NDs of Domains 9, 10 and 12, as can be seen in Table 3.

**Table 3.** Nursing diagnoses with risk of mortality in critical patients with COVID-19 – Rio Branco, AC, Brazil, 2020–2021.

Nursing diagnoses*	n	%	Hazard ratio	p-value
**DOMAIN 1. Health promotion**				
Ineffective protection	41	100.0	5.22	0.01
Frailty syndrome	24	45.3	1.83	0.03
**DOMAIN 2. Nutrition**				
Imbalanced nutrition: lower than the required	30	100	2.76	0.12
Obesity	17	94.5	1.77	0.05
Risk of impaired liver function	33	100.0	1.18	0.68
Unstable blood glucose risk	36	100.0	1.78	0.78
Risk of fluid imbalance	29	100.0	1.92	0.26
**DOMAIN 3. Elimination and exchange**				
Constipation	36	94.8	2.14	0.46
Diarrhea	40	97.5	1.83	0.39
**DOMAIN 4. Activity/exercise**				
Impaired ambulation	6	100.0	1.93	0.44
Impaired physical mobility	8	100.0	1.88	0.85
Decreased cardiac output	19	95.0	1.78	0.04
Ineffective breathing pattern	55	100.0	1.88	0.02
Ineffective peripheral tissue perfusion	25	100.0	4.71	0.03
Impaired spontaneous ventilation	19	100.0	1.78	0.04
Deficit in bathing self-care	4	100.0	3.46	0.08
**DOMAIN 5. Perception/cognition**				
Acute confusion	44	97.7	6.2	0.02
Impaired verbal communication	13	100.0	4.2	0.03
DOMAIN 9. Coping/tolerance**				
Anxiety	7	77.7	5.30	0.07
DOMAIN 10. Life principles**				
Impaired religiosity	2	66.7	1.04	0.30
**DOMAIN 11. Safety/protection**				
Risk of infection	26	100.0	2.75	0.09
Aspiration risk	9	90.0	1.93	0.44
Risk of shock	20	95.3	0.57	0.67
Risk of corneal injury	14	93.3	5.05	0.08
Risk of pressure injury	33	97.0	2.39	0.30
Contamination	20	100.0	3.95	0.02
Hyperthermia	30	96.7	1.56	0.45
Hypothermia	44	95.6	1.85	0.39
DOMAIN 12. Comfort**				
Risk of loneliness	2	66.7	1.58	0.45
Social isolation	2	100.0	0.34	0.90

*May have more than one diagnosis. *Missings.

## DISCUSSION

Data from history taking and admission physical examination revealed that patients with COVID-19, admitted to this ICU, were extremely severe and dependent on Nursing care. The Nursing Diagnoses with the highest risk of mortality were: Ineffective protection; Ineffective tissue perfusion; Contamination; Ineffective Breathing Pattern; Impaired spontaneous ventilation; Acute confusion; Frailty syndrome; Obesity, and Decreased cardiac output. Identifying clinical indicators and NDs of higher mortality risk helps in clinical observation and in early nursing and interdisciplinary interventions^(15)^.

Many patients were over 60 years of age and had comorbidities, risk factors for COVID-19 mortality^(7,8,11,13)^. In addition, brown and black patients, common characteristics of patients in the North region of Brazil, were the majority among those hospitalized with severe cases in the ICU^(11.16)^, whose unit was established in a few days due to the lack of intensive care beds in the state with a simpler technological structure when compared to large centers, which leads to a social discussion in the COVID-19 pandemic^(11,16,17,18,19)^.

In a multicenter cohort study carried out in California, high-tech hospital admission was a protective factor against unfavorable outcomes, unlike the ICU of the study, whose socioeconomic disparities can interfere with survival and deserve intervention and political-sanitary discussion^(16–19)^.

Regarding the clinical indicators presented by the patients in the study, related to the higher risk of mortality from COVID-19, the following stand out: dyspnea, fever, fatigue, cough, increased heart rate, arrhythmias, and obesity. These manifestations are defining characteristics of the NDs IBP, Impaired spontaneous ventilation, Decreased cardiac output, Ineffective tissue perfusion, Contamination and Ineffective Protection, which shall undergo early surveillance for these symptoms and signs and referral for Nursing interventions^(5,9,11,13,20,21)^.

The ND Ineffective protection refers to the decrease in the ability to protect oneself from internal or external threats characterized by the presence of dyspnea, fatigue, and cough^(4)^. This diagnosis has, as populations at risk, extremes of age, reaffirming the results obtained in the research in which older patients are more vulnerable to mortality, and should be the priority in preventive practices such as vaccination against COVID-19^(5,9,20,21)^.

The RePPE suggested, as Nursing activities for the NDs Ineffective tissue perfusion and Decreased cardiac output, Shock Control, Medication Administration, Hydroelectrolytic Control, and Hemodynamic Regulation, with the nursing prescriptions Monitoring and vital signs assessment, Control of urinary output and fluid balance, Observation and reporting in case of systolic blood pressure being lower than 90 mmHg and of the presence of arrhythmias on the monitor, perfusion status (extremity temperature, skin color) assessment, as well as recording and evaluation of Central Venous Pressure, Pulmonary Artery Pressure, Pulmonary Capillary Pressure, Cardiac Output, and Venous Oxygen Saturation, if monitoring catheter installed, and Administration of fluids according to institutional protocol^(3,5,9)^.

Ineffective Breathing Pattern (IBP) is an ND characterized by inspiration and/or expiration that does not provide adequate ventilation; it is related to fatigue, pain, obesity, and anxiety, and is characterized by the presence of dyspnea, tachypnea, and abnormal breathing pattern^(5)^. This ND had a 100.0% frequency and an 88.0% higher risk for mortality in patients with COVID-19. In a study in the same ICU, but without the presence of COVID-19, the frequency of this ND was 66.7%, revealing a large increase in the population of patients with COVID-19^(11)^.

The ND Impaired spontaneous ventilation is the progression of the clinical worsening of patients diagnosed with IBP, in which the patient is unable to maintain spontaneous ventilation, requiring ventilatory support and, in most cases, caused by respiratory muscle fatigue^(5)^. These patients have to be evaluated at the bedside by the nurse, who shall assess respiratory rate, measure and control fluid balance, acid-base disorder, perform pulmonary auscultation, monitor signs of level of consciousness lowering, among others, always discussing with the multidisciplinary team, which will help in the prescription of drugs and in respiratory physiotherapy, aiming at improving the respiratory condition and the respiratory muscles fatigue^(5,9,15,20,21)^.

The ND Deficit in self-care for bathing and feeding are very frequent in critically ill ICU patients, due to the patient’s inability to perform hygiene and feeding measures correctly. In COVID-19 patients, specific care is recommended for bed baths, such as disposable baths, oral hygiene with oxygen peroxide or povidone in conscious and oriented patients and with 0.12% chlorhexidine, every 12 hours, in intubated patients; also, giving preference to oil-free moisturizers and using a protocol for prone positioning^(5,22)^.

The Risk of Pressure Injury is conceptualized as the susceptibility to skin and/or adjacent tissue injury, usually on the bony prominence due to pressure, and has as risk factors inadequate nutrition, the impossibility of changing positions due to the severe clinical condition, self-care deficit, and patients with extreme age^(5)^. A very high incidence was identified in the same ICU, in the period from 2012 to 2014, 42.7%^(23)^, much higher than that of an ICU in Vale do São Francisco, in Pernambuco, where a prevalence of 22.3% was identified and associated with the patient’s origin from the emergency room and hospitalization time equal to or greater than ten days^(24)^. In this regard, as the incidence of the diagnosis Risk for Pressure Injury was high, it is essential that the patient with COVID-19, especially in the prone position, is provided with preventive measures as the use of cushions in areas that were not usual for prevention, such as the face, ears, knees, among others^(9,22,24)^. In addition, a diet focused on the patient’s needs, dialoguing with the nutritionist and the physician, and risk and lesion progression assessment through specific scales, such as Braden’s. Studies have revealed that malnutrition severity increases the severity and likelihood of developing pressure injuries^(25)^.

Another ND present is the Risk of corneal injury, which, although not statistically significant for mortality, has drawn attention to eye care in these patients^(5,22,26–28)^. In a previous cohort study in Acre, in a non-COVID ICU, the incidence of corneal injury was 18.8%, which demonstrates that one out of five patients had corneal injury, which is considered a high incidence^(26)^.

The risk of corneal injury is very common in ICU patients due to changes in the mechanism of blinking and closing the eyelids caused by the level of consciousness lowering and the use of sedatives and muscle blockers^(5,26–28)^. Nursing eye care includes eye hygiene with gauze and saline solution, lubrication of the corneas with eye drops or lubricating ointments and manual eyelid closure in case of lagophthalmos. Moreover, scientific evidence suggests that the use of a gel lubricant and a polyethylene chamber are the best evidence to prevent corneal injury in ICU patients^(27,28)^.

The patients’ clinical records presented little information of NDs of domains 9, 10 and 12, such as Anxiety, Fear, and Impaired Religiosity, which may also reflect the professional team’s emphasis on the biomedical model, requiring further continuing education on patients’ biopsychosocial assessment, which aims to understand them in their entirety^(5,29)^.

As limitations, the study had a small sample due to an organizational failure by SAME, from where the data were collected. This failure occurred due to the great demand from the pandemic, which made the finding of other clinical records difficult. There was also lack of information in the medical records to establish the NDs of domains 9, 10 and 12, whose observation infers the need for education in the health service, aiming to make professionals attentive to the patients’ psychosocial and spiritual aspects.

However, even with the limitations, data were collected by a nursing resident in the intensive care unit. Furthermore, this study is among the first Brazilian projects with clinical validation of ND in critically ill patients with COVID-19 and was the first to analyze the diagnoses of higher risk of mortality, configuring care based on the principles of the scientific method, aiming to direct the nursing care planning for early care of critically ill patients with COVID-19.

## CONCLUSION

This study infers the need to monitor the clinical indicators dyspnea, fever, fatigue, cough, increased heart rate, arrhythmias, and obesity, which will signal the Nursing Diagnoses with a higher risk of mortality, namely, Ineffective protection, Ineffective tissue perfusion, Contamination, Ineffective Breathing Pattern, Impaired spontaneous ventilation, Acute confusion, Frailty syndrome, Obesity, and Decreased cardiac output in critical COVID-19 patients.

## ASSOCIATE EDITOR

Marcia Regina Cubas
